# Three-dimensional ultrastructural analysis of cells in the periodontal ligament using focused ion beam/scanning electron microscope tomography

**DOI:** 10.1038/srep39435

**Published:** 2016-12-20

**Authors:** Shingo Hirashima, Keisuke Ohta, Tomonoshin Kanazawa, Satoko Okayama, Akinobu Togo, Naohisa Uchimura, Jingo Kusukawa, Kei-ichiro Nakamura

**Affiliations:** 1 Division of Microscopic and Developmental Anatomy, Department of Anatomy, Kurume University School of Medicine, Kurume 830-0011, Japan; 2 Dental and Oral Medical Center, Kurume University School of Medicine, Kurume 830-0011, Japan; 3 Cognitive and Molecular Research Institute of Brain Diseases, Kurume University School of Medicine, Kurume, 830-0011, Japan; 4 Electron Microscopic Laboratory, Central Research Unit of Kurume University, Kurume 830-0011, Japan

## Abstract

The accurate comprehension of normal tissue provides essential data to analyse abnormalities such as disease and regenerative processes. In addition, understanding the proper structure of the target tissue and its microenvironment may facilitate successful novel treatment strategies. Many studies have examined the nature and structure of periodontal ligaments (PDLs); however, the three-dimensional (3D) structure of cells in normal PDLs remains poorly understood. In this study, we used focused ion beam/scanning electron microscope tomography to investigate the whole 3D ultrastructure of PDL cells along with quantitatively analysing their structural properties and ascertaining their orientation to the direction of the collagen fibre. PDL cells were shown to be in contact with each other, forming a widespread mesh-like network between the cementum and the alveolar bone. The volume of the cells in the horizontal fibre area was significantly larger than in other areas, whereas the anisotropy of these cells was lower than in other areas. Furthermore, the orientation of cells to the PDL fibres was not parallel to the PDL fibres in each area. As similar evaluations are recognized as being challenging using conventional two-dimensional methods, these novel 3D findings may contribute necessary knowledge for the comprehensive understanding and analysis of PDLs.

An accurate understanding of normal tissue is important toward providing the baseline data necessary to effectively analyse abnormalities such as disease and regenerative processes. In particular, three-dimensional (3D) data are useful for linking structure to function and dysfunction^1^,^2^. Recently, novel tissue engineering methodologies have been developed termed bottom-up tissue engineering, which aims to address the challenges of recreating biomimetic structures by designing structural features on the microscale to build modular tissues that can in turn be used as building blocks to create larger tissues^3^,^4^,^5^. Understanding the proper structure of the target tissue and controlling the microenvironment may facilitate successful novel treatment strategies, as organ function involves biological cooperation with the surrounding tissues and other organs^6^,^7^.

The periodontal ligament (PDL) is a connective tissue consisting primarily of collagen fibre bundles (collagen types I and III) and cells found between the roots of teeth and the inner walls of the alveolar socket. The cells of the PDL are heterogeneous including fibroblasts, osteoblasts, cementoblasts, and stem cells^8^. The PDL firmly anchors the tooth to the bone via Sharpey’s fibres and distributes applied force to contiguous alveolar bone. The PDL thus represents an essential tissue that maintains the periodontal environment and function and is therefore important in dentistry including in both periodontics and orthodontics. A comprehensive understanding of the organization and function of the PDL might contribute to the elucidation of several biological processes including alveolar bone remodelling mechanisms involving orthodontic force, mechanisms of root resorption, and regeneration of periodontal tissue. Furthermore, studies of the PDL might also contribute to the development of safe orthodontic treatments, effective regeneration therapies, and new oral implants containing PDL-like tissue.

Many studies have examined the nature and structure of PDLs; for example, the direct 3D visualization of the collagen network and of the changes that occur when the tooth is loaded has been reported^9^. Therefore, the use of novel 3D analysis methods might provide additional new insight. In particular, fibroblasts are known to have important roles in the synthesis of collagen bundles in the PDL. Additionally, fibroblasts of the PDL are interconnected by gap- and adherence-type junctions^8^,^10^,^11^ and have been suggested to form a highly structured and interconnected network^8^. In single sections, it is recognized that PDL cells are oriented parallel to the collagen bundles. However, the 3D ultrastructure of these PDL cells, which synthesize collagen fibre as an essential component of the PDL, is poorly understood. In addition, there are no reports related to the 3D ultrastructure of the cellular architecture among PDLs.

In order to elucidate the nature of the 3D architecture of PDLs, it is necessary to observe detailed whole images of PDL fibres and cells in 3D using electron microscopy (EM). However, it is difficult to evaluate whole images in detail using conventional light microscopy (LM) or EM as both cells and collagen bundles are organized on a mesoscale^12^,^13^.

Recently, a new method for serial sectioning and 3D analytical scanning electron microscopy has been developed, termed focused ion beam/scanning electron microscope tomography (FIB/SEM tomography)^14^,^15^,^16^. FIB/SEM enables the observation of hundreds of serial sections and FIB/SEM tomography enables the reconstruction of 3D structures with high resolution in addition to the quantitative analysis of structural properties. We previously utilized FIB/SEM tomography to investigate the structure of organelle, cell, and collagen bundles in various tissues^16^,^17^,^18^,^19^,^20^,^21^.

In the current study, we have expanded these analyses to investigate the whole 3D ultrastructure of PDL cells using FIB/SEM tomography and additionally quantitatively analyse the structural properties of PDL cells and the correlation between cells and collagen bundles.

## Results

### Single section analysis by LM and EM

**Figure 1:**
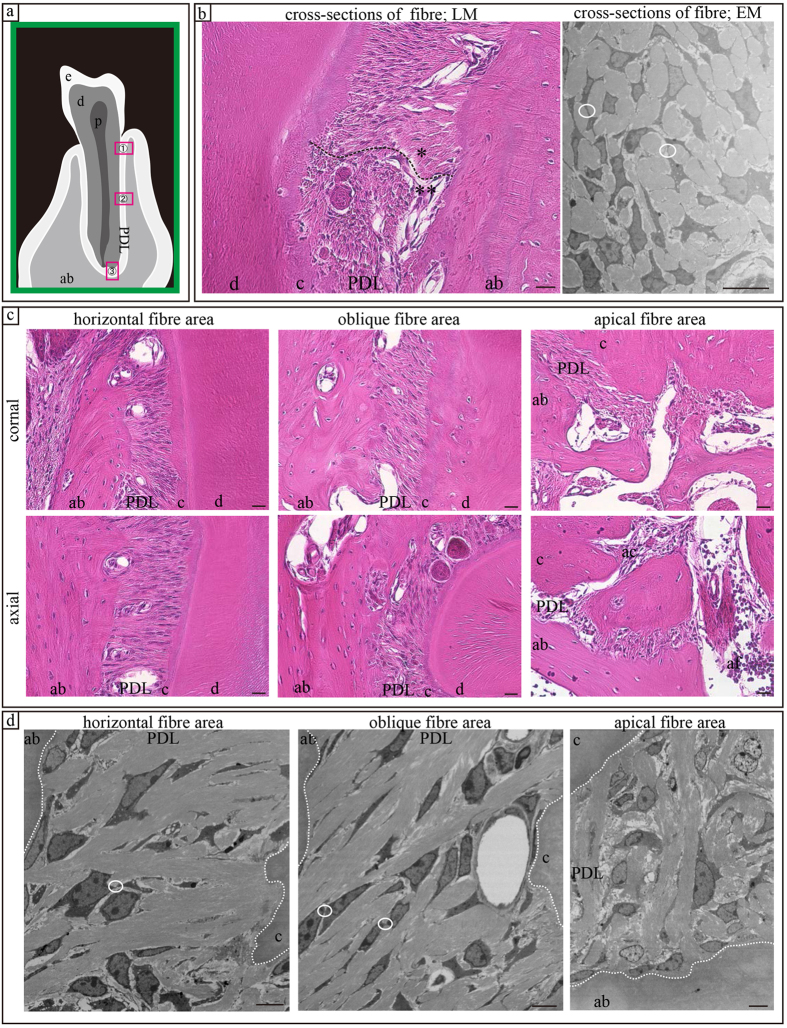
Single sections imaged using plain light (LM) and electron microscopy (EM). (**a**) Schematic diagram of the observation area: (1) horizontal fibre area, (2) oblique fibre area, and (3) apical fibre area. (**b**) Cross section of a fibre using LM and EM. In LM, the fibres were in parallel sections (*) or cross-sectioned (**). (**c**,**d**) In the axial and coronal sections, PDL cells were oriented parallel to fibres. ab, alveolar bone; PDL, periodontal ligament; e, enamel; d, dentin; c, cementum; af, apical faramen; ac, accessory canal. Scale bars: 20 μm for EM images, 10 μm for LM images.

In cross-sections of collagen bundles (Fig. 1b), the cells exhibited a stellate appearance when observed using LM and EM. Cell processes segregated individual collagen bundles whereas cytoplasmic processes were connected, forming a mesh-like network. PDL fibres were present in the network.

In coronal and axial sections imaged by LM (Fig. 1c), PDL cells appeared to be oriented parallel to the PDL as previously reported^8^. However, only the outline of the cells was observed and detailed morphology could not be evaluated. PDL fibres were transversely oriented between the root cementum and the socket wall.

In EM (Fig. 1d), detailed cellular morphology could be clearly ascertained. The cells were spindle shaped and exhibited oval nuclei and a smooth surface. Processes were observed between the PDL fibres. PDL cells were in contact with each other in all areas (Fig. 1d) and were oriented parallel to the fibres.

### FIB/SEM tomography: serial cross-sections

**Figure 2:**
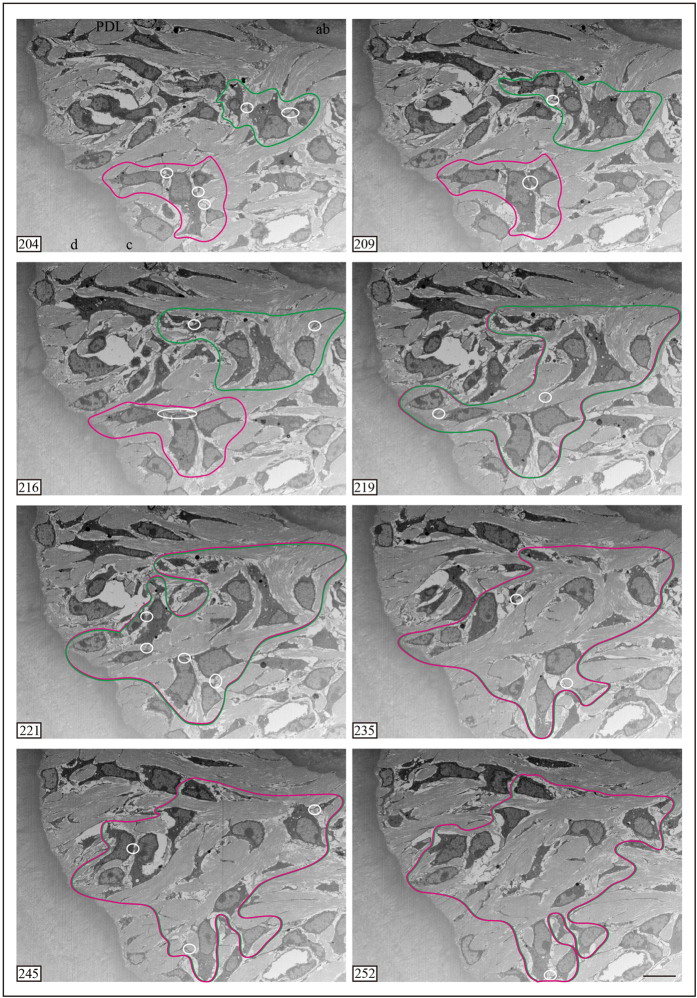
Serial cross-sections imaged using FIB/SEM tomography. Magenta and green lines represent regions with contacted cells. White circles and ellipses represent cell contacts. Magenta and green regions in slices 204–216 are separate areas. The two areas in slice 219 overlap and the overlapping region is expanded. ab, alveolar bone; PDL, periodontal ligament; e, enamel; d, dentin; c, cementum. Scale bars: 10 μm for all panels.

**Figure 3:**
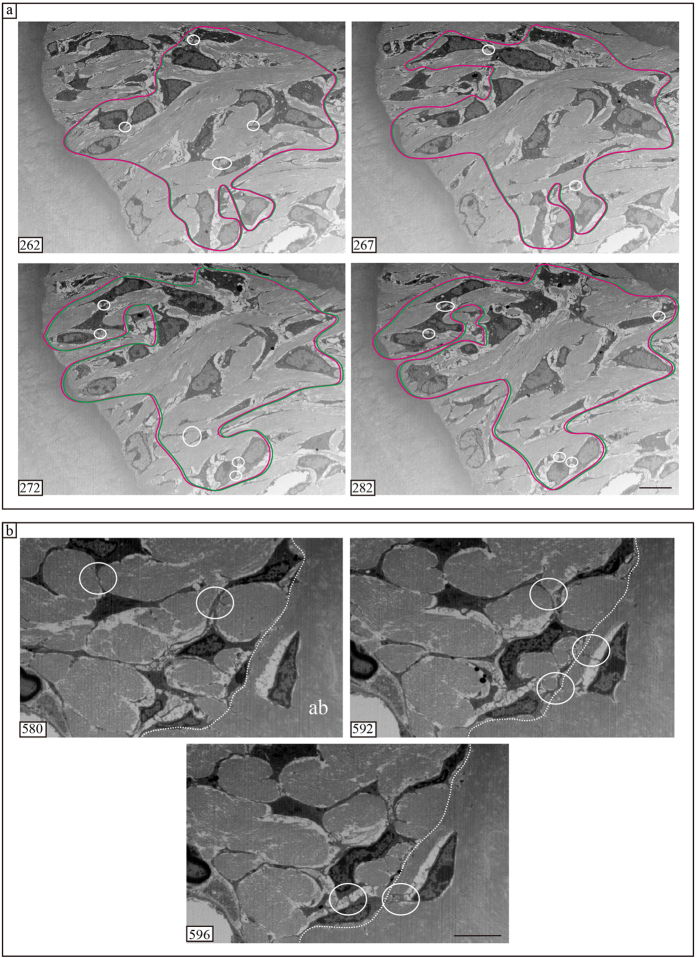
Serial cross-sections imaged using FIB/SEM tomography. (**a**) Magenta and green lines represent regions with contacted cells. White circles and ellipses represent cell contacts. The overlapping region is large, leading from the alveolar bone to the cementum. PDL cells form a widespread cellular network. (**b**) PDL cells are in contact with other PDL cells and with osteoblast-like cells. Osteoblasts-like cells were in contact with osteocyte -like cells (white circles in slices 580, 592, and 596). ab, alveolar bone. Scale bars: 10 μm for panel (a), 5 μm for panel (b).

When viewed using FIB/SEM, PDL cells were observed to be in contact with each other (Figs 2 and 3a). In single sections, the area of the cell that was in direct contact with other cells was narrow (Figs 2 and 3a). Conversely, in serial cross-sections, areas of the cell that were in direct contact overlapped at different regions (Fig. 2). Notably, the cell contact region was large, ranging from the alveolar bone to the cementum (Fig. 3a). Thus, although in single sections the cells did not appear to be in contact because of the extensive long distance between the cells, in serial cross-sections contacts were observed between the cells. Additionally, PDL cells were in contact with both osteoblasts-like and osteocytes-like cells (Fig. 3b, Supplementary movie S1).

### FIB/SEM tomography: 3D-structure reconstruction

**Figure 4:**
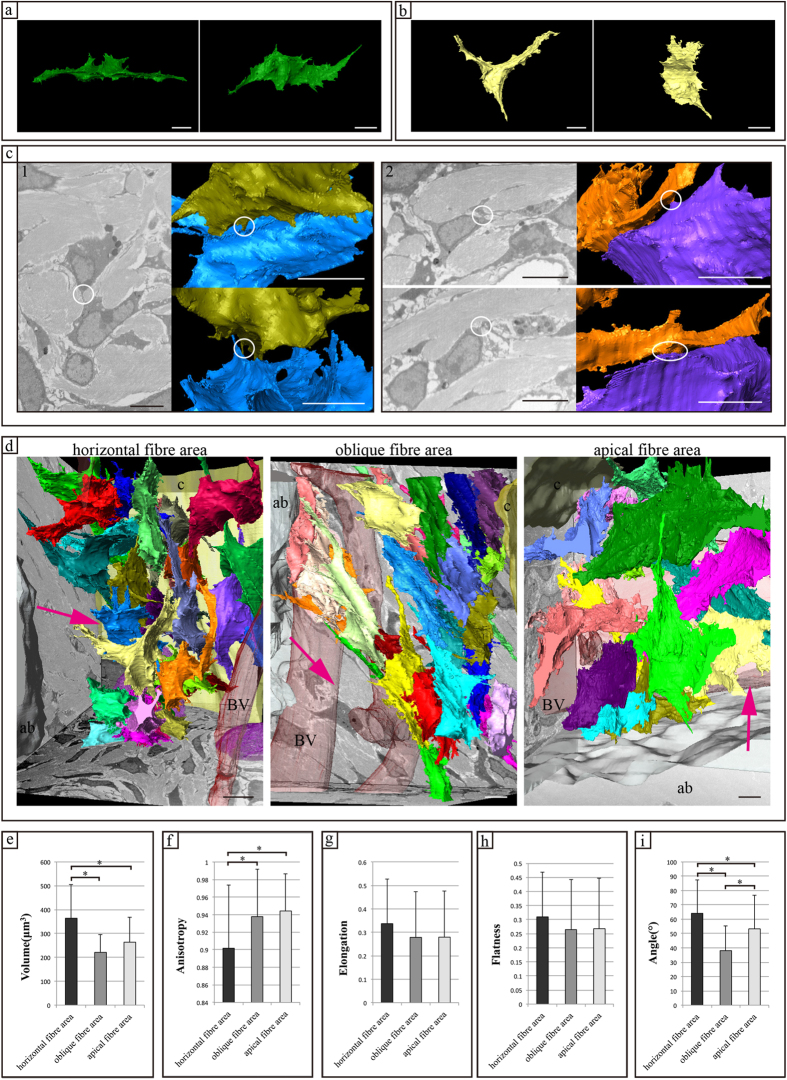
Three-dimensional structure and structural properties of PDL cells. (**a**,**b**) Reconstructed images of PDL cells are shown. PDL cells exhibit a flat shape with long processes, showing a wing-like rather than a spindle shape. Fine processes are observed on the cell surface. (**c**) Reconstructed images of contacts between cells. The contacts are (1) point to point or (2) end to end within the white circle. (**d**) Reconstructed images of PDL cells. Magenta arrows show the direction of PDL fibres. The arrays of cells and PDL fibres differ depending on the observed area. (**e**–**h**) Results of quantitative analysis of the cell morphology as assessed by FIB/SEM tomography. (**i**) Angles between each cell and the collagen bundle. The histogram shows the means for each group and the error bars represent standard deviations. The asterisk indicates a significant difference (P < 0.05). ab, alveolar bone; PDL, periodontal ligament; e, enamel; d, dentin; c, cementum; BV, blood vessel. Scale bar: 5 μm for all panels.

Next, we reconstructed 3D images based on the FIB/SEM tomography data. In each part of the PDL, cells had a flat shape with long processes and exhibited a wing-like rather than a spindle-like shape (Fig. 4a,b). PDL cells were in contact with neighbouring cells in either a point-to-point or end-to-end orientation (Fig. 4c1,2). PDL cells formed a widespread mesh-like network between the cementum and alveolar bone (Fig. 4d, Supplementary Movie S2) and PDL fibres were present among the cellular network.

In the horizontal, oblique, and apical fibre areas, the average volumes of PDL cells were 363.7 ± 142.02, 220 ± 75.40, and 263 ± 104.20 μm^3^, respectively. The volume of PDL cells in the horizontal fibre was significantly different from those of the other areas (P < 0.05 for all) (Fig. 4e).

In the horizontal, oblique, and apical fibre areas, the average anisotropies of the cells were 0.90 ± 0.07, 0.93 ± 0.05, and 0.94 ± 0.04, respectively. In contrast, the anisotropy of PDL cells in the horizontal fibre was significantly different from those of the other areas (P < 0.05) (Fig. 4f). No differences were found in the elongation or flatness of the PDL cells among the three areas (Fig. 4g,h).

The orientation of cells to the PDL fibres differed depending on the observation area. In the horizontal fibre area, PDL cells tended to orient with the longitudinal axis at right angles to the direction of the PDL fibres, with an average angle of 64.0 ± 23.48°. In the oblique fibre area, PDL cells and fibres tended to be arranged in parallel to the direction of PDL fibres, where the average angle was 38.0 ± 17.23°. In the apical fibre area, the PDL cells exhibited a random orientation to the PDL fibres, with an average angle of 53.2 ± 23.38°. Thus, there were significant differences among the three groups (P < 0.05 for each comparison) (Fig. 4i).

## Discussion

In the present study, we first clarified the 3D ultrastructure of PDL cell morphology using FIB/SEM tomography. We showed that PDL cells from the tooth to the alveolar bone formed a widespread cellular network, and also elucidated the structural properties of PDL cells such as their whole shape and the orientation of the cells to the PDL fibres themselves. In previous studies, the surfaces of PDL fibres were observed with SEM after treatment with a chemical reagent. Furthermore, although PDL fibres and cells can be observed in detail using single sections, 3D architecture analysis of the PDL by reconstruction of serial single sections is difficult. FIB/SEM tomography was considered the preferred method for observing histological 3D structure without being confounded by methodology or artefacts.

In the current study, we demonstrated that PDL cells contacted with each other and formed a widespread cellular network between the cementum and the alveolar bone. Furthermore, we showed that PDL cells were in contact with osteoblast- and osteocyte- like cells. Cellular networks have been observed in several tissues and organs including the bone, colon, and cornea, wherein the involvement of cellular networks with tissue function is strongly indicated^22^,^23^,^24^,^25^,^26^,^27^,^28^. Additionally, it has been suggested that fibroblasts regulate osteoblast behaviour partially through gap junctions^29^. Therefore, cells in the PDL and bone are expected to form widespread, complex cellular networks that may contribute to the structural basis of cell-to-cell collaborations and to various cellular activities. However, the detailed junction type could not be determined in the present study. Previously, it was reported that fibroblasts of the PDL were interconnected by gap- and adherence-type junctions^8^, which suggests that the contacts in our observation may also represent these junction types. Our findings suggest that widespread cellular networks might function synchronously via the gap junctions and might therefore be responsible for the synthesis of collagen fibre and the metabolic activities essential for the maintenance of PDL fibres over the total distance between the teeth and the alveolar bone.

We also found that the orientation of cells relative to the PDL fibres differed depending on the observation area. Dartsch and Hammerle^30^ reported that cells subjected to mechanical stimulation show a high degree of orientation and that the cellular array is related to the direction of stretching. The angle of cell orientation varies in direct relation to the stretching amplitude and becomes steeper in correlation to the intensity of the mechanical stimulus. Thus, Dartsch and Hammerle^30^ suggested that a certain threshold is required to induce cell response and orientation. The cell shape was also shown to change when the collagen bundles were rearranged by tensile force^31^, with spreading and bipolar elongation of the cells and their processes occurring along the aligned collagen. In particular, Qian *et al*.^32^ reported that the strain was concentrated in the PDL with the maximum tensile strain located at the sides of the tooth roots, whereas the maximum compressive strain was located at the tooth apices based on finite element analysis. This study indicates that the distribution of the strain in the PDL was not uniform. The cell orientation findings of the present study might therefore represent an important factor that determines the direction of fibres and cells in each area of the PDL.

We analysed the morphology including anisotropy, elongation, flatness, and volume of PDL cells. Notably, we demonstrated that the shape of the cells upon 3D observation differed from that upon the 2D interpretation. It is well known that PDL cells mainly consist of fibroblasts^8^ and conventional 2D observations previously showed that fibroblasts appeared as spindle shaped^23^. Conversely, in our present study, PDL cells presented a flat shape with long processes exhibiting a wing-like appearance. We have also reported that fibroblasts may exhibit a sheet-like shape as well^19^. Furthermore, the term ‘fibroblasts’ can apply to a variety of cell populations^23^ and fibroblasts play several roles^33^,^34^,^35^,^36^; for example, in wound healing, tissue repair, fibrosis, and metastases. Thus, our findings indicate that fibroblasts exhibit diverse 3D morphologies and multiple functions. However, although the volume and anisotropy in the horizontal fibre area demonstrated significant differences from those in other areas, we were unable to obtain data sufficient to determine the specific cell types in this region. Therefore, for the detailed classification of cell types in the PDL, molecular biological techniques are required.

The present study had several limitations. First, these specimens were obtained from rodents. To improve our understanding of the ultrastructure of the PDL, it is necessary to analyse tissues from various species, particularly humans, using FIB/SEM tomography. Second, our sample size using FIB/SEM tomography was limited to 100 × 100 × 100 μm^3^ areas. To obtain a better understanding of the entire architecture and function of the PDL, additional methods such as micro-computed tomography, array tomography, or deep imaging method are required in combination with FIB/SEM tomography for future studies. These methods may allow the analysis of data from large animal studies such as in canine or porcine models as well as the dynamic analysis of tooth-PDL-bone complexes in orthodontic force application models. Finally, this is a comparative study of the regional morphology among normal PDL. Therefore, we did not utilize any application models to alter or regenerate these cells. However, we believe that our findings would benefit future studies that intend to evaluate changes of the PDL by manipulating mechanical stress via various force applications.

In conclusion, we present the first observation of the 3D ultrastructure of the PDL as shown by FIB/SEM tomography. Accordingly, we revealed the presence of widespread cellular networks, the orientation of cells relative to the PDL fibres in each observed area, and demonstrated the 3D shape of the PDL cells and that they were not parallel to the PDL fibres. Together, these findings provide a deeper understanding of the architecture of the PDL, which in turn may contribute to the elucidation of changes in the microenvironment during the process of tooth movement, the regeneration process, and the development of more effective and novel treatment strategies especially in the field of dentistry.

## Materials and Methods

All experiments were performed in accordance with the National Institutes of Health Guidelines for animal research. All animal procedures were approved by the Board for Animal Experiments of Kurume University.

### Observation area

The PDL region at the centre of the mesial root of the lower first molar was divided into three areas (Fig. 1a): 1) horizontal fibre area, 2) oblique fibre area, and 3) apical fibre area. Each area was observed by LM or EM.

### Tissue preparation for LM specimens

Twenty-four-week-old male C57BL/6 mice (n = 5) were deeply anesthetized with diethyl ether and sodium pentobarbital (30 mg/kg) and were transcardially perfused through the left ventricle with heparin (10 U/ml) in saline, followed by fixation with 4% paraformaldehyde in phosphate-buffered saline (PBS). After perfusion, the mandible containing the teeth and PDL was removed from the skull. The specimens were immersed in the same fixative for 2 hours at 4 °C and were then decalcified in Kalkitox solution (Wako Pure Chemical Industries, Ltd., Osaka, Japan) for 5 days. The specimens were then embedded in paraffin. Paraffin blocks were cut into 6-μm-thick sections using a microtome (HM340E; Thermo Scientific, Waltham, MA, USA) in the axial and coronal planes. The sections were stained with haematoxylin and eosin and observed.

### Tissue preparation of EM specimens

Specimens were prepared as described previously^16^,^19^. Male C57Bl/6 mice (n = 3) were deeply anesthetized with diethyl ether and sodium pentobarbital (30 mg/kg) and transcardially perfused through the left ventricle with heparin (10 U/ml) in saline, followed by fixation with 2% paraformaldehyde and incubation in 2.5% glutaraldehyde in 0.1 M cacodylate (pH 7.3) buffer for EM. After perfusion, the mandible was removed from the skull. The specimens were immersed in the same fixative for 2 hours at 4 °C and then washed in buffer and decalcified as described above. After decalcification, the specimens were washed in buffer three times for 10 minutes each.

Next, specimens were cut into small cubes and subjected to postfixation and *en bloc* staining. Briefly, after three washes in cacodylate buffer, the specimens were postfixed for 2 hours in a solution containing 2% osmium tetraoxide and 1.5% potassium ferrocyanide in cacodylate buffer at 4 °C. The specimens were then washed three times with distilled water and immersed in 1% thiocarbohydrazide solution for 1 hour. After five washes with distilled water, the specimens were further immersed in 2% osmium tetraoxide in distilled water and then washed three times with distilled water. The specimens were stained *en bloc* in a solution of 4% uranyl acetate dissolved in distilled water overnight for contrast enhancement and then washed with distilled water. Next, the specimens were further stained with Walton’s lead aspartate solution for 2 hours^37^, dehydrated in an ethanol series (25%, 50%, 70%, 80%, 90%, and twice in 100% ethanol for 5 minutes each), infiltrated with epoxy resin (Epon 812; TAAB, Aldermaston, UK), and polymerized for 72 hours at 60 °C. The surfaces of the embedded specimens were exposed using a diamond knife on an Ultracut E microtome (Leica, Wetzlar, Germany). The resin blocks were then trimmed down and placed on a holder.

### FIB/SEM tomography and 3D-structure reconstruction

Serial images of the block faces were acquired by repeated cycles of sample surface milling and imaging using an FIB/SEM apparatus (Quanta 3D FEG, FEI, Eindhoven, The Netherlands) and Slice & View G2 operating software (FEI). Milling was performed with a gallium ion beam at 30 kV with a current of 15 nA. The milling pitch was set to 100 nm/step and 800 cycles. The images were acquired at a landing energy of 2.5 keV. Additional acquisition parameters were as follows: beam current = 50.8 pA, dwell time = 6 μs/pixel, image size = 2048 × 1768 pixels, and pixel size = 36 nm/pixel. The resulting image stack, segmentation, and 3D reconstruction were processed using Fiji software (http://fiji.sc/Fiji) and Amira 6.0.1 software (FEI Visualization Science Group, Burlington, MA, USA). Images were observed with optional X-Y-Z plane sectioning.

After reconstruction, we performed quantitative analysis of whole cells contained within the observation range. In total, 106 cells were analysed, including 36 cells in the horizontal fibre area, 34 cells in the oblique fibre area, and 36 cells in the apical fibre area. The angle between each cell and the collagen bundle was measured. Using the Amira software, sections containing the vector of each cell and the vertical aspect to the direction of a fibre were obtained. Each of the normal vectors was then calculated. To measure the angle of each cell compared with the direction of the fibre, the inner product between the normal vectors of each cell and the direction of the fibre was calculated. Additionally, the volume, anisotropy, elongation and flatness of PDL cells were calculated. Anisotropy was determined as 1 minus the ratio of the smallest to the largest eigenvalue, with spherical objects yielding small values close to 0. Elongation was determined as the ratio of the medium to the largest eigenvalue, with elongated objects giving small values close to 0. Flatness was determined as the ratio of the smallest to the medium eigenvalue, with flat objects being assigned small values close to 0.

### Statistical analysis

Statistical analysis was performed using JMP version 11 (SAS Institute Inc.). Kruskal-Wallis tests with Wilcoxon post-hoc analyses were used to evaluate three areas by comparing volume, anisotropy, elongation, flatness, and angle between cells and the collagen bundle. Values are shown as the means ± standard deviations (SDs). Differences with P values of less than 0.05 were considered significant.

## Additional Information

**How to cite this article**: Hirashima, S. *et al*. Three-dimensional ultrastructural analysis of cells in the periodontal ligament using focused ion beam/scanning electron microscope tomography. *Sci. Rep.* **6**, 39435; doi: 10.1038/srep39435 (2016).

**Publisher's note:** Springer Nature remains neutral with regard to jurisdictional claims in published maps and institutional affiliations.

## Supplementary Material

Supplementary Information

Supplementary Movie S1

Supplementary Movie S2
