# Supplementary material for: Three-dimensional ultrastructural analysis of cells in the periodontal ligament using focused ion beam/scanning electron microscope tomography
Source: Sci Rep. 2016 Dec 20;6:39435. doi: 10.1038/srep39435 (PMC5171660; doi:10.1038/srep39435)
Supplement: Supplementary Information [file srep39435-s1.pdf]

Three-dimensional ultrastructural analysis of cells in the periodontal ligament using focused ion beam/scanning electron microscope tomography

Shingo Hirashima[1, 2, 4], Keisuke Ohta[1, 3], Tomonoshin Kanazawa[1], Satoko Okayama[1], Togo Akinobu[1, 3], Naohisa Uchimura[4], Jingo Kusukawa[2], Kei-ichiro Nakamura[1]

[1]Division of Microscopic and Developmental Anatomy, Department of Anatomy, Kurume University School of Medicine, Kurume 830-0011, Japan

[2]Dental and Oral Medical Center, Kurume University School of Medicine, Kurume 830-0011, Japan

[3]Electron Microscopic Laboratory, Central Research Unit of Kurume University, Kurume 830-0011, Japan

[4]Cognitive and Molecular Research Institute of Brain Diseases, Kurume University School of Medicine, Kurume, 830-0011, Japan.

Corresponding author: Shingo Hirashima, e-mail: hirashima_shingo@med.kurume-u.ac.jp

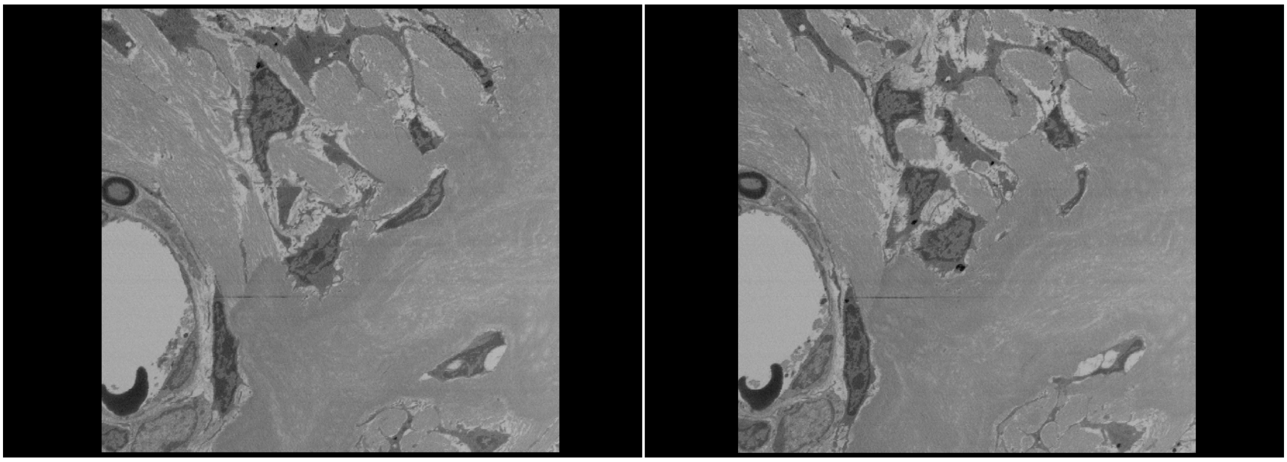

Supplementary movie S1. Animation of serial cross-sections imaged using FIB/SEM tomography in Figure 3b. PDL cells are in contact with each other. Additionally, PDL cells are in contact with both osteoblast-like and osteocyte-like cells.

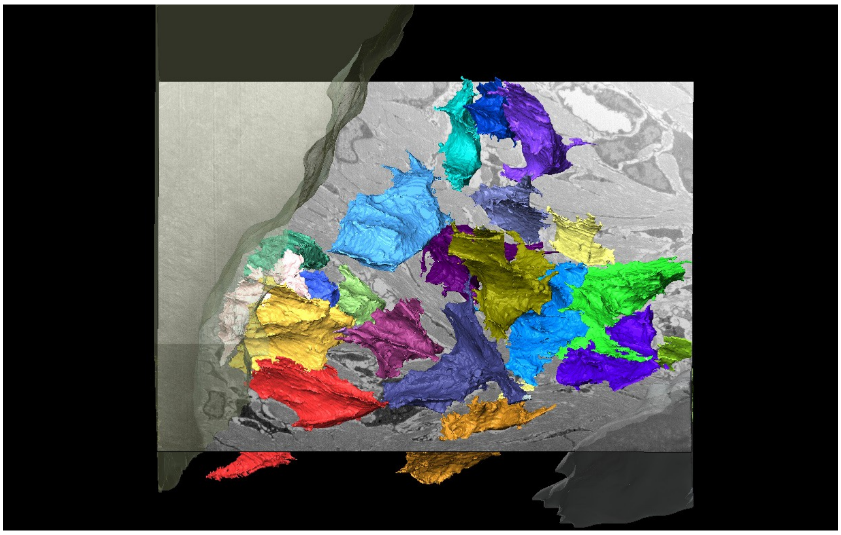

Supplementary movie S2. Animation of the PDL cellular network in Figures 2 and 3a. PDL cells are in contact with neighboring cells and form a widespread mesh-like network between the cementum and alveolar bone. Additionally, reconstructed images of PDL cells are shown. PDL cells are flat with long processes, showing a wing-like and not a spindle-like shape.
